# GEFT Inhibits Autophagy and Apoptosis in Rhabdomyosarcoma *via* Activation of the Rac1/Cdc42-mTOR Signaling Pathway

**DOI:** 10.3389/fonc.2021.656608

**Published:** 2021-06-18

**Authors:** Chunsen Li, Zhenzhen Li, Lingxie Song, Lian Meng, Guixuan Xu, Haijun Zhang, Jianming Hu, Feng Li, Chunxia Liu

**Affiliations:** ^1^ Department of Pathology and Key Laboratory for Xinjiang Endemic and Ethnic Diseases, Shihezi University School of Medicine, Shihezi, China; ^2^ Department of Pathology and Medical Research Center, Beijing Chaoyang Hospital, Capital Medical University, Beijing, China; ^3^ Department of Pathology, The Second Affiliated Hospital of Guangzhou Medical University, Guangzhou, China

**Keywords:** autophagy, apoptosis, Rac1, Cdc42, mTOR

## Abstract

Autophagy and apoptosis are dynamic processes that determine the fate of cells, and regulating these processes can treat cancer. GEFT is highly expressed in rhabdomyosarcoma (RMS), which accelerates the tumorigenicity and metastasis of RMS by activating Rac1/Cdc42 signaling, but the regulatory mechanisms of autophagy and apoptosis are unclear. In our study, we found that the RMS tissues had high Rac1, Cdc42, mTOR, and Bcl-2 expression levels and low Beclin1, LC3, and Bax expression levels compared with the normal striated muscle tissues (*P* < 0.05). In addition, multivariate analysis has proven that Rac1 is an independent prognostic factor (*P* < 0.05), and the high expression level of the Beclin1 protein was closely associated with the tumor diameter of the RMS patients (*P* = 0.044), whereas the high expression level of the LC3 protein was associated with the clinical stage of the RMS patients (*P* = 0.027). Furthermore, GEFT overexpression could inhibit autophagy and apoptosis in RMS. A Rac1/Cdc42 inhibitor was added, and the inhibition of autophagy and apoptosis decreased. Rac1 and Cdc42 could regulate mTOR to inhibit autophagy and apoptosis in RMS. Overall, these studies demonstrated that the GEFT–Rac1/Cdc42–mTOR pathway can inhibit autophagy and apoptosis in RMS and provide evidence for innovative treatments.

## Introduction

Rhabdomyosarcoma (RMS) is a common malignant tumor in adolescents and children with soft tissue tumors. It can be divided into alveolar RMS (ARMS), embryonic RMS (ERMS), and polymorphic RMS (PRMS) (1). Despite current multimodal treatments, patients with recurrent or metastatic disease still remain in poor condition, and new therapies are required to improve the efficacy of RMS treatment (2). Regulating autophagy and promoting tumor cell death has recently become a new approach to tumor treatment (3). Thus far, few studies have shown that the dysregulation of programmed cell death (apoptosis and autophagy) is closely related to the metastasis and formation of RMS (4, 5). Therefore, research on the mechanism of autophagy and autophagy regulation may provide new strategies for the treatment of RMS.

GEFT, a guanine nucleotide exchange factor that promote the release of GDP bound to the GTPase, allowing the binding of a GTP molecule (6), is highly expressed in muscles and is closely related to tumorigenesis, invasion, and metastasis (7). Our previous experiments have confirmed that GEFT is highly expressed in RMS and associated with survival and prognosis (8). In addition, GEFT leads to metastasis and tumorigenicity of RMS by activating EMT induced by Rac1/Cdc42 signaling (9). We have also identified that GEFT is regulated by the microRNA-29 family to inhibit the formation and progression of RMS (10) and is directly targeted by miR-874 to decrease the proliferation, invasion, migration, and anti-apoptotic ability of RMS (11). However, the potential pathways and functions of GEFT in autophagy and apoptosis of RMS are unknown.

Rac1 is a widely expressed member of the GTPase family, which plays an important role in many cancer-related signaling processes. Rac1 can substantially inhibit the proliferation of primary schwannoma cells by inducing apoptosis (12). Deacetylmycoepoxydiene can drive Rac1 activation, promote the production of reactive oxygen species, and simultaneously induce autophagy and apoptosis in lung cancer (13). Rac1 prevents UV-induced keratinocyte apoptosis by regulating the DNA damage response in skin cancer (14). Cdc42 is a small GTPase associated with a variety of human cancers, and it is related to cell cycle progression, migration/invasion, tumor growth, and oncogenic transformation. One study has suggested that Cdc42 may be a molecular regulator of the autophagy response to the tumor microenvironment (15).

In addition, some studies have shown that mTOR can affect autophagy and apoptosis in tumor cells through various pathways (16, 17). YAP inhibits autophagy-associated apoptosis in hepatocellular carcinoma through the Rac1–mTOR pathway (18). Simvastatin enhances autophagy by inhibiting the Rac1–mTOR signaling pathway in coronary myocardial cells (19). However, only a few studies have been reported on the effect of autophagy and apoptosis on the expression or activity of RMS cells, and their role in the progression of RMS is unclear. GANT-61 (GLI1/2 inhibitor) can inhibit tumor cell proliferation and block tumor growth in ARMS and ERMS animal models by inhibiting the Shh/AKT–mTOR signaling axis (20). NVP-BEZ235 (PI3K/mTOR inhibitor) and chloroquine can play a synergistic role in inducing the apoptosis of ERMS cells (21).

Here, our study has demonstrated that Rac1, Cdc42, p-mTOR, and Bcl-2 proteins are highly expressed in RMS tissues, whereas Beclin1, LC3, and Bax are expressed at low levels in RMS tissues. GEFT can inhibit the expression of autophagy and apoptosis in RMS cell lines and transplanted tumor tissues. Interestingly, Rac1, Cdc42, and an mTOR inhibitor can also promote the autophagy and apoptosis of overexpressed GEFT. Therefore, GEFT can inhibit autophagy and apoptosis in RMS by regulating the Rac1/Cdc42–mTOR pathway, providing new insights into the pathogenesis of RMS and developing new therapeutic strategies.

## Materials and Methods

### Tissue Samples

A total of 62 formalin-fixed paraffin-embedded RMS and 20 normal striated muscle tissue samples were selected from the archives of the Department of Pathology, the First Affiliated Hospital of Shihezi University Medical College and the First Affiliated Hospital of Xinjiang Medical University, China. All participating patients submitted a written informed consent. This study was conducted in accordance with the ethical guidelines of the Helsinki Declaration and approved by the Institutional Ethics Committee of the First Affiliated Hospital of Shihezi University School of Medicine.

### Tissue Microarrays

A representative paraffin-embedded tissue block was obtained from the patients for the experiments. The original hematoxylin and eosin sections were reviewed, and tissue microarrays were established from the tumor-representative areas of the paraffin-embedded tissue blocks. A representative area (3 mm in diameter) was collected from each paraffin block and arranged in a tissue array by using a ring drill. Finally, the tissue chip (4 μm thick) was subjected to immunohistochemical staining.

### Cell Culture and Transfection

Our study used two human RMS cells, including RH30 (ARMS) and RD (ERMS) cell lines (Shanghai Fuxiang Biotechnology, China). We selected RMS cells and RMS cells that overexpressed GEFT constructed by lentivirus transfection. All the cells were cultured in DMEM (GIBCO, USA), 10% FBS (BI, Israel), and 10% penicillin streptomycin (Solarbio, China) at 37°C and 5% CO_2_. The cells were transfected with Lipofectamine 2000 (Life Technologies, USA). 2×10^5^ cells were inoculated into each hole in the 6-well plate, and 1μg/ml polybrene was added when the number of cells reached about 50-70%. The virus solution needed for virus infection was absorbed according to the MOI value, and the culture medium was changed after 16 hours, and the culture medium was changed after 16 hours. 24 hours after infection, the cells were screened with predetermined puromycin, and the fluorescence expression was observed 72 hours after infection for follow-up experiments.

### Antibodies and Inhibitors

The main antibodies used for immunohistochemistry (IHC) were as follows: mouse anti-Rac1 (Ab33186, 1:800; Abcam), mouse anti-Cdc42 (Ab187643, 1:200; Abcam), rabbit anti-p-mTOR (#2974, 1:250; Cst), rabbit anti-Beclin1 (Ab55878, 1:200; Abcam), rabbit anti-LC3A/B (#12741, 1:100; Cst), mouse anti-Bax (Ab32503, 1:800; Abcam), rabbit anti-Bcl-2 (Ab112, 1:1000; Beyotime), and rabbit anti-caspase-3 (#9662, 1:1000; Cst).

The main antibodies and inhibitors used for Western blot were as follows. The primary antibodies were rabbit anti-GEFT (Ab127690, 1:1000; Abcam), rabbit anti-mTOR (Ab32028, 1:1000; Abcam), rabbit anti-p-mTOR (#2974,1:1000; Cst), rabbit anti-Beclin1 (Ab55878, 1:1000; Abcam), rabbit anti-LC3A/B (#12741,1:1000; Cst), mouse anti-Bax (Ab32503, 1:1000; Abcam), rabbit anti-Bcl-2 (Ab112, 1:1000; Beyotime), rabbit anti-caspase-3 (#9662,1:1000; Cst), rabbit anti-cleaved-PARP (#32064, 1:1000; Cst), and mouse anti-β-actin (IE9A3, 1:800; China). The secondary antibody was peroxidase-conjugated goat anti-mouse/rabbit IgG (ZB-2305, 1:10000; ZSGB). Rac1 Activation Assay Biochem KitTM (cytoskeleton, Cat. # BK035) and Cdc42 Activation Assay Biochem KitTM (cytoskeleton, Cat. # BK034) were used for analysis of Rac1 and Cdc42 activation.

The NSC23766 (S8031, 50 μM/mL, Selleck), ZCL278 (S7293, 100 μM/mL, Selleck) and Rapamycin (S1039, 100 μM/mL, Selleck) were added to the cells and treated for 48 hours. CQ (HY-17589A, 1.6 μM/mL, MCE), BafA-1 (HY-10058, 0.4 μM/mL, MCE) were added to the cells and treated for 24 hours. Rac1 Activation Assay Biochem KitTM (cytoskeleton, Cat. # BK035) and Cdc42 Activation Assay Biochem KitTM (cytoskeleton, Cat. # BK034) were used for analysis of Rac1 and Cdc42 activation. All inhibitors were acquired commercially, which were used in various cellular functional experiments and WB experiments.

### Immunohistochemical Staining

The sections were dewaxed and hydrated, and the antigen was repaired. The slides and antibodies were incubated overnight in a blocking solution at 4°C. On the second day of incubation, the slides and primary antibodies were incubated with a secondary antibody, instilled with DAB, and restaining with hematoxylin, and finally dehydration. The tumor sections previously identified as positive were included in each staining procedure to ensure consistency in the immunohistochemical assessment.

### Immunohistochemical Scoring

The criterion for identification was the determination of staining in the tissues based on the immune response score proposed by Remmele and Stegner (22). Tumors and normal striated muscle were semi-quantitatively assessed in accordance with the percentage of positive cells and the intensity of cytoplasmic staining. The proportion of positive staining scores was as follows: 0 (≤ 5%), 1 (6%–25%), 2 (26%–50%), and 3 (≥ 51%). The intensity of staining was as follows: 0 (negative), 1 (buff), 2 (yellow), and 3 (brown). The dyeing index was calculated using the following formula: dyeing index = dyeing intensity × dyeing grade. Therefore, the staining results were classified as follows: − (0), + (1–3), ++ (4–6), and +++ (7–9), where − indicates a negative expression, and +, ++, and +++ represent positive expression. All staining results were independently evaluated by two pathologists who did not know the patients.

### RNA Extraction and Quantitative Real-Time PCR (qRT-PCR)

The total RNA was extracted from cultured cells or human samples by using a paraffin wax RNA extraction kit (Omega Bio-Tek, USA). The total RNA was reverse transcribed into cDNA by using the QuantiTect Reverse Transcription Kit (QIAGEN, Germany). The qRT-PCR analysis was carried out using the Quanti Fast TM SYBR Green PCR Kit (QIAGEN, Germany) and 7500 Real-Time Fluorescence PCR System (Applied Biosystems, USA). The PCR primers were designed on the basis of the gene sequences of human Rac1, Cdc42, and β-actin (Shenggong, China). The sequences of the β-actin forward and reverse primers were 5′-AGCACAGAGCCTCGCCTTTG-3′ and 5′-ACATGCCGGAGCCGTTGT-3′, respectively. The sequences of the Rac1 forward and reverse primers were 5′-CCGGTGAATCTGGGCTTATG-3′ and 5′-CTCGGATCGCTTCGTCAAAC-3′, respectively. The sequences of the Cdc42 forward and reverse primers were 5′-CAGGTGTGTGCTGCTATGAACATC-3′ and 5′-GTAGGTGCAGGGCATTTGTCATTA-3′, respectively. The relative expression levels of Rac1, Cdc42, and β-actin were normalized using the 2^−ΔΔCt^ method.

### Western Blot

Protein concentration was determined through the BCA method by using RIPA lysis buffer (Solarbio) in accordance with the manufacturer’s requirements. An equal amount of protein (20 μg) was applied to a 10% gel for electrophoresis and transferred onto a PVDF membrane (Solarbio). The membrane was transferred to a blocking solution for 2 h on a shaking bed at room temperature, incubated with the primary antibody at 4°C overnight, and then incubated with the secondary antibody for 2 h at room temperature. Finally, the membrane was visualized using the ECL Luminescence Assay Kit (Biyuntian Biotechnology). Western blot density was assessed using the ImageJ 1.46 software.

### Co-Immunoprecipitation (Co-IP)

The transfected RMS cells were lysed in RIPA lysis buffer, and the protein lysate was centrifuged. The target and IgG antibodies were added in equal proportions in accordance with the antibody instructions. After incubating the protein and antibody for 12 h, agarose beads (50 µL) were added and shaken at 4°C for 12 h. The agarose beads were collected, added to the loading buffer in equal proportions, and boiled at 95°C for 5 min to break the bond between the agarose beads and the protein. The supernatant was collected and placed in a dry bath at 100°C, boiled for 10 min, cooled to room temperature, and stored in a freezer at −20°C until Western blot analysis.

### Immunofluorescence

The cells with a concentration of 2×10^5^ cells/ml were fixed in a culture plate with 2% paraformaldehyde solution and washed with PBS. Then, the cells were permeated with 2 mL of 0.2% – 0.5% Triton XMel 100 for 10 min and cleaned with PBS. After sealing with 2% BSA for 30 min, an antibody (1:1000) was added and incubated overnight in the dark. The next day, after washing with PBS, the second antibody (1:10000) was added and incubated for 45 min; 0.5 µg/mL of DAP was added for staining for 10 min, and PBS was washed. Finally, the cells were observed and photographed under a microscope.

### Monodansylcadaverine (MDC) Labeling

After the cells reached the logarithmic growth phase, the concentration was adjusted to 2×10^5^ cells/ml, the cultured cells were centrifuged for 5 min and then washed with 300 μL of 1×wash buffer. 1× Wash buffer resuspension was added to the cells, and the cell concentration was adjusted to 2×10^6^ mL. Ninety microliters of cell suspension were added with 10 μL of MDC stain (Solarbio, China), stained at room temperature, and stored in the dark for 45 min. Then, the cells were centrifuged, and 100 μL of collection buffer was added to resuscitate cells. The treated cells were dripped on the slide, and the cells were observed and photographed under the fluorescence microscope (the wave length of the excitation filter was 355 nm, and the wavelength of the blocking filter was 512 nm).

### Acridine Orange (AO) Staining

Adjust the concentration of well-growing cells to 2×10^5^ cells/ml. The culture medium of the treated RMS cells was taken out and washed with 1×PBS. The cells were stained with 1 mg/mL acridine orange and 1 mg/mL propidium iodide (Solarbio, China), and then incubated in the dark for 15 minutes. The stained cells were observed and photographed under a microscope.

### TUNEL Staining

The terminal deoxynucleotidyl transferase dUTP nick end labelling (TUNEL) Apoptosis Detection Kit was obtained from Shanghai Biyuntian Biotechnology. NSC23766 (50 μM/mL, Rac1 inhibitor), ZCL278 (100 μM/mL, Cdc42 inhibitor) and Rapamycin (100 μM/mL, mTOR inhibitor) were added when the cells grew to 50% confluency. After 48 h, the cells were fixed with 500 μL of 4% paraformaldehyde (Solarbio) and incubated with a highly permeable immunostaining liquid for 5 min at room temperature. TUNEL detection solution (50 μL) was added to each well, followed by incubation at 37°C for 1 h, after which DAPI was added and then incubated for 5 min. The apoptotic rate was determined under a fluorescence microscope, and statistical analysis was performed.

### Flow Cytometry Analysis of Apoptosis

The RH30 and RD cells were grown in cell culture flasks. Adherent cells were detached using 0.025% trypsin and fixed in 2% paraformaldehyde. These cells were washed in phosphate-buffered solution, collected after centrifugation, and incubated in PBS for 5 min on ice. The cell concentration was adjusted to 2×10^5^ cells/ml, and the cells in each group were mixed with 5 μl Annexin V–FITC/PI. The cells were Stained for 5-15 min and detected within 1 hour. The analysis was performed using PAS flow cytometry (PARTEC, Germany) and FlowJo 7.6 software.

### Animal Studies

The animal study was approved by the Ethics Committee of the first affiliated Hospital of Shihezi University Medical College. Five-week-old male nude mice were randomly divided into four groups: RH30 (RD) + GEFT group, RH30 (RD) + inhibitor group. According to the standard scheme of QIAGEN, lentivirus was used to construct RH30 and RD cells. Subcutaneous injection of 2×10^6^ RH30 or RD cells was stably transfected with a GEFT overexpression lentiviral vector. After the nude mice formed a tumor, the nude mice in each group were weighed every 2 days, and the size of the subcutaneous tumor was measured. When the diameter of the tumor reached 0.5 cm, NSC23766 (50 mg/kg) or ZCL278 (50 mg/kg) was added to the RH30 (RD) - GEFT + NSC23766 group or RH30 (RD) - GEFT + ZCL278 group, with 5 nude mice in each group. After 2 weeks of inhibitor treatment, the nude mice were killed, and the tumors were extracted. The tumor specimens were fixed in 4% neutral formaldehyde solution for 24 h, and the sections were prepared for Western blot. some transplanted tumor tissues were taken from each nude mice for IHC staining.

### Statistical Analysis

The data were expressed as the average ± standard deviation. Statistical significance was assessed by comparing the means and independent group t-tests. Statistical analysis was performed using the Kaplan–Meier, χ^2^ or Fisher’s exact test, log-rank tests, and the Cox proportional hazard model. All experiments were repeated at least three times. The data were analyzed using SPSS 20.0 software. ***represents P < 0.001, **represents P < 0.01, *represents P < 0.05. *P* < 0.05 was considered as statistically significant.

## Results

### Rac1, Cdc42, and p-mTOR Are Expressed at High Levels in RMS Tissues, Whereas Autophagy and Apoptosis-Related Proteins Are Expressed at Low Levels in RMS Tissues

**Figure 1:**
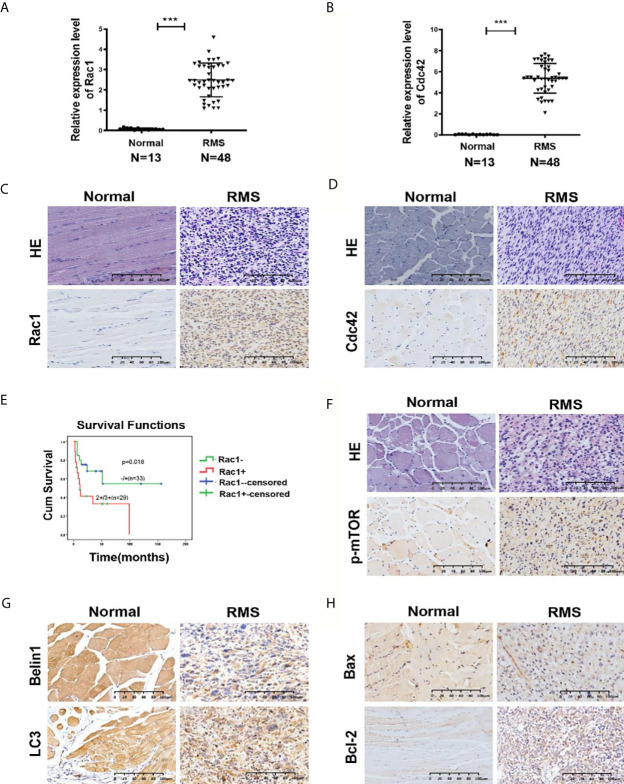
Expression of Rac1, Cdc42, p-mTOR, and autophagy- and apoptosis-related molecules in RMS tissues. **(A, B)** Quantitative real-time PCR (qRT-PCR) was used to detect the mRNA expression levels of Rac1 **(A)** and Cdc42 **(B)** in 48 RMS cases and 13 normal muscle tissue cases. **(C, D)** HE and IHC staining of Rac1 **(C)** and Cdc42 **(D)** in normal and RMS tissues. **(E)** Kaplan–Meier analysis and log-rank test were applied to determine the relationship between Rac1 protein expression and patient survival. **(F)** HE and IHC staining of p-mTOR in normal and RMS tissues. **(G)** IHC staining of Beclin1 and LC3 in normal and RMS tissues. **(H)** IHC staining of Bax and Bcl-2 in normal and RMS tissues. A representative image is provided. ***P < 0.001.

Our previous studies have revealed that GEFT is highly expressed in RMS and associated with disease stage and metastasis, which promotes RMS cell survival, invasion, and migration by activating the Rac1/Cdc42 pathway (8, 9). A total of 48 RMS cases and 13 normal striated muscle tissue cases were assessed using qRT-PCR to investigate the expression levels of Rac1 and Cdc42 in RMS and explore whether Rac1 and Cdc42 were highly expressed in RMS. The expression levels of Rac1 and Cdc42 mRNA in RMS (2.399 ± 6.52989 and 5.317 ± 16.0144, respectively) were significantly higher than those in normal muscle tissues (0.1262 ± 0.20052 and 0.033 ± 0.5756, respectively; *P* < 0.001; **Figures 1A, B**). The protein expression levels of Rac1 and Cdc42 were detected using IHC. The results showed that the rate of Rac1 protein in RMS was 89% (55/62), whereas the rate of Rac1 expression in the 20 normal muscle tissue samples was 65% (13/20, **Table 1**). Compared with the controls, the positive expression rate differences were statistically significant (χ^2^ = 4.446, *P* = 0.035; **Figure 1C**). The rate of Cdc42 protein expression in the RMS and normal control samples was 83% (19/23) and 55% (11/20), respectively (**Table 2**). A significant difference in Cdc42 expression was observed between the tumor and normal tissues (χ^2^ = 3.866, *P* = 0.049; **Figure 1D**).

**Table 1:** Expression of Rac1 and p-mTOR protein in different types of RMS and normal muscle tissue.

Tissue type	N	Rac1(%)		χ^2^	*P*	p-mTOR(%)		χ^2^	*P*
		Negative	Positive			Negative	Positive		
RMS	62	7 (12.9)	55 (87.1)	4.446	0.035	14 (22.6)	48 (77.4)	5.492	0.019
ARMS	18	0 (0)	18 (100)			2 (11.1)	16 (88.9)		
ERMS	29	6 (20.7)	23 (79.3)			7 (24.1)	22 (75.9)		
PRMS	8	1 (12.5)	7 (87.5)			3 (37.5)	5 (62.5)		
Other	7	5 (71.4)	2 (28.6)			2 (28.6)	5 (71.4)		
Control	20	7 (35.0)	13 (65.0)			10 (50)	10 (50)		

Others represent nonspecific types of RMS. Control represents normal muscle tissue.

**Table 2:** Expression of Cdc42 protein in different types of RMS and normal muscle tissue.

Tissue type	N	Cdc42		χ^2^	*P*
		Negative	Positive		
RMS	23	4 (17.39)	19 (82.6)	3.866	0.049
ARMS	9	1 (11.11)	8 (88.9)		
ERMS	10	2 (20)	8 (80.0)		
PRMS	4	1 (25)	3 (75)		
Control	20	9 (45)	11 (55)		

Control represents normal muscle tissue.

The survival prognosis analysis of the Rac1 protein showed that the survival rate of patients with high Rac1 expression levels was significantly lower than that of patients with low Rac1 expression levels (*P* = 0.018, **Figure 1E**). The high expression level of the Rac1 protein was closely related to the tumor site of the RMS patients (*P* = 0.025, **Supplementary Table S1**). The expression level (*P* = 0.026), age (*P* = 0.033) and TNM stage (*P* = 0.014) of the Rac1 protein could influence the survival prognosis of RMS (**Supplementary Table S2**). Cdc42 and p-mTOR were also studied, and no significant correlation was observed with various clinicopathological parameters. Correlation analysis showed that the protein expression of GEFT was positively correlated with that of Rac1 (r = 1.000, P < 0.001), Cdc42 (r = 1.000, P < 0.001), p-mTOR (r = 0.548, P < 0.012), and Bcl-2 (r = 0.795, P = 0.001), but negatively correlated with LC3 protein expression (r = -0.428, P = 0.05, **Supplementary Table S3**).

We continued to verify whether autophagy and apoptotic molecules were involved in the occurrence of RMS. The rate of p-mTOR protein expression in RMS and control was 77% (48/62) and 50% (10/20), respectively (**Table 1**). A significant difference in p-mTOR expression was observed between the tumor and normal tissues (χ^2^ = 5.492, *P* = 0.019; **Figure 1F**). The rate of Beclin1 protein expression in RMS and normal muscle samples was 73% (45/62) and 100% (20/20), respectively. The rate of LC3 protein-positive expression in RMS and normal muscle samples was 69% (43/62) and 95% (19/20), respectively (**Supplementary Table S4**). Compared with the controls, the Beclin1- and LC3-positive expression rate differences were statistically significant (χ^2^ = 5.350, *P* = 0.021 and χ^2^ = 4.092, *P* = 0.043; respectively, **Figure 1G**). The high expression level of the Beclin1 protein was closely associated with the tumor diameter of the RMS patients (*P* = 0.044, **Supplementary Table S5**), whereas the high expression level of the LC3 protein was associated with the clinical stage of the RMS patients (*P* = 0.027, **Supplementary Table S6**). The rate of Bax protein expression in RMS and normal muscle samples was 53% (16/30) and 100% (15/15), respectively. The rate of the Bcl-2 protein expression in RMS and normal muscle samples was 93% (28/30) and 33% (5/15), respectively (**Supplementary Table S7**). Compared with the controls, the Bax and Bcl-2-positive expression rate differences were statistically significant (χ^2^ = 8.101, *P* = 0.004 and χ^2^ = 15.469, *P* < 0.001, respectively, **Figure 1H**). Therefore, all the mentioned results demonstrated that Rac1, Cdc42, p-mTOR, and Bcl-2 were expressed at high levels in RMS tissues, whereas Beclin1, LC3, and Bax were expressed at low levels in RMS tissues.

### GEFT Can Inhibit Autophagy and Apoptosis in RMS Cells

**Figure 2:**
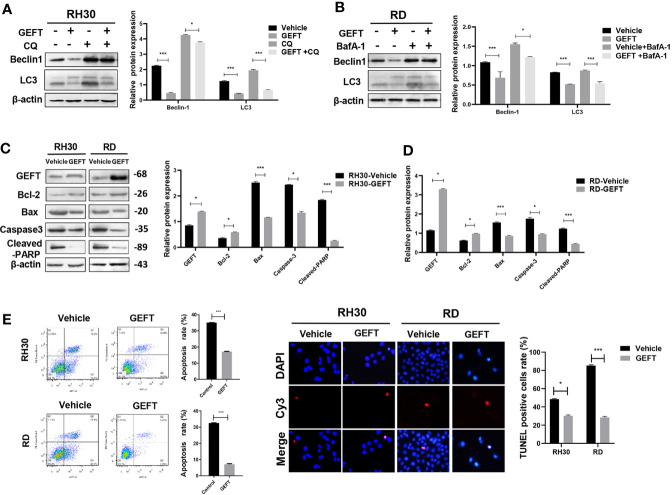
GEFT affects the autophagy and apoptosis in RMS cells. **(A)** Western blotting was used to detect the expression of Beclin1 and LC3 protein in RH30 cells for control groups, control+CQ groups, transfected with GEFT, transfected with GEFT+CQ. **(B)** Western blotting was used to detect the expression levels of Beclin1 and LC3 protein in RD cells for control groups, control+BafA-1 groups, transfected with GEFT groups, transfected with GEFT+ BafA-1 groups. **(C)** Western blotting was used to detect the expression levels of GEFT, Bax, Bcl-2, Caspase-3, and Cleaved-PARP proteins in RH30 and RD cells transfected with the GEFT and control groups. **(D)** Apoptosis of RH30 and RD cells transfected with GEFT and control group was detected by flow cytometry. **(E)** Apoptosis of RH30 and RD cells transfected with the GEFT and control groups was detected by TUNEL. ***P < 0.001 and *P < 0.05.

In order to explore the relationship between GEFT and autophagy and apoptosis, we chose to detect autophagy and apoptosis in cells under the intervention of GEFT. The Western blot results showed that the expression of autophagy-related proteins, namely, Beclin1 and LC3 in RMS cells decreased after stable transformation of GEFT (*P* < 0.001 and *P* < 0.05). When autophagy inhibitors, namely, CQ and BafA-1, were added, the expression of Beclin1 and LC3 proteins decreased after stable transformation of GEFT (*P* < 0.001 and *P* < 0.05; **Figures 2A, B**). In addition, the Western blot results demonstrated that the expression levels of apoptosis-related proteins, namely, Bax, Caspase3, and Cleaved-PARP, decreased in RMS cells after the stable conversion of GEFT, whereas the expression level of the Active Rac1, Active Cdc42 and Bcl-2 proteins increased (*P* < 0.05 and *P* < 0.001, **Figure 2C**). The rate of apoptosis in RH30 and RD cells after the stable GEFT transformation was reduced compared with that of the control group (*P* < 0.001, **Figure 2D**). The TUNEL results showed that apoptosis was reduced after the overexpression of GEFT (**Figure 2E**). These results suggested that the overexpression of GEFT may inhibit autophagy and apoptosis in RMS cells.

### Regulation of Rac1 and Cdc42 by GEFT Can Inhibit Autophagy and Apoptosis in RMS Cells

**Figure 3:**
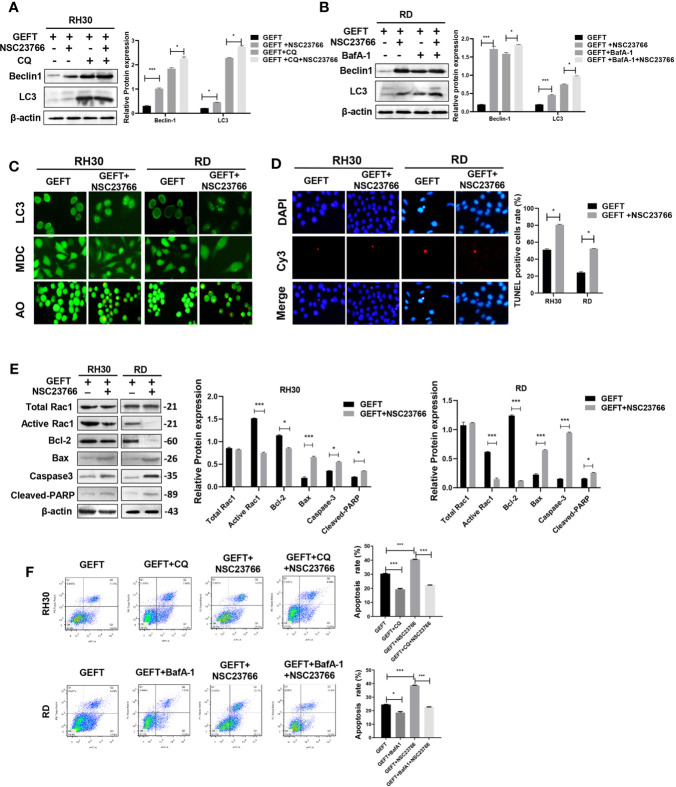
GEFT can inhibit autophagy and apoptosis in RMS cells *via* Rac1. **(A)** Western blotting was used to detect the expression of Beclin1 and LC3 protein in RH30 cells transfected with GEFT, GEFT+NSC23766, GEFT+CQ, and GEFT+CQ+NSC23766. **(B)** Western blotting was used to detect the expression of Beclin1 and LC3 protein in RD cells transfected with GEFT, GEFT+NSC23766, GEFT+BafA-1, and GEFT+BafA-1+NSC23766. **(C)** The representative images of RH30 and RD cells transfected with GEFT and GEFT+NSC2376 were detected by immunofluorescence, MDC and AO staining. **(D)** The representative images of RH30 and RD cells transfected with GEFT and GEFT+NSC2376 were detected by TUNEL staining. **(E)** Western blotting was used to detect the expression of GEFT, Total Rac1, Active Rac1, Bax, Bcl-2, Caspase-3, and Cleaved-PARP protein in RH30 and RD cells transfected with GEFT and GEFT+NSC23766. **(F)** Flow cytometry was used to detect the apoptosis of RH30 cells and GEFT-transfected RH30 cells in GEFT, GEFT+CQ, GEFT+NSC23766 and GEFT+NSC23766+CQ groups. Flow cytometry was used to detect the apoptosis of RD cells and GEFT-transfected RD cells in GEFT, GEFT+BafA-1, GEFT+NSC23766 and GEFT+NSC23766+BafA-1 groups. ***P < 0.001, *P < 0.05.

**Figure 4:**
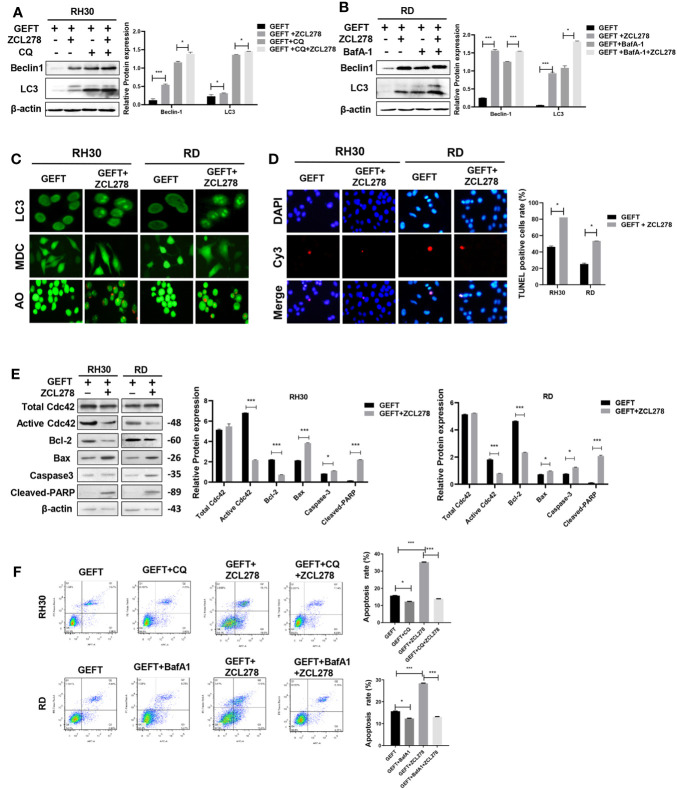
GEFT can inhibit autophagy and apoptosis in RMS cells *via* Cdc42. **(A)** Western blotting was used to detect the expression of Beclin1 and LC3 protein in RH30 cells transfected with GEFT, GEFT+ZCL278, GEFT+CQ, and GEFT+CQ+ZCL278. **(B)** Western blotting was used to detect the expression of Beclin1 and LC3 protein in RD cells transfected with GEFT, GEFT+ZCL278, GEFT+BafA-1, and GEFT+BafA-1+ZCL278. **(C)** The representative images of RH30 and RD cells transfected with GEFT and GEFT+ZCL278 were detected by immunofluorescence, MDC and AO staining. **(D)** The apoptosis of RH30 and RD cells transfected with GEFT and GEFT+ZCL278 was detected by TUNEL. **(E)** Western blotting was used to detect the expression of GEFT, Total Cdc42, Active Cdc42, Bax, Bcl-2, Caspase-3, and Cleaved-PARP protein in RH30 and RD cells transfected with GEFT and GEFT+ZCL278. **(F)** Flow cytometry was used to detect the apoptosis of RH30 cells and GEFT-transfected RH30 cells in GEFT, GEFT+CQ, GEFT+ZCL278 and GEFT+ZCL278+CQ groups. Flow cytometry was used to detect the apoptosis of RD cells and GEFT-transfected RD cells in GEFT, GEFT+BafA-1, GEFT+ZCL278 and GEFT+ZCL278+BafA-1 groups. ***P < 0.001, *P < 0.05.

GEFT can promote the invasion and migration of RMS cells through Rac1/Cdc42 (9). A Rac1 inhibitor (NSC27366) was added to RMS cells and RMS cells stably transfected with GEFT lentivirus to verify whether GEFT inhibits autophagy and apoptosis through Rac1/Cdc42. The results showed that the expression of Beclin1 and LC3 protein in the NSC23766 group was significantly higher than that in the normal untreated group, and the expression levels of Beclin1 and LC3 protein in the NSC23766 group also increased after adding CQ and BafA-1 (*P* < 0.05 and *P* < 0.001, **Figures 3A, B**). In addition, immunofluorescence showed that the dot pattern of LC3 fluorescence was clearly observed in the NSC23766 group. MDC staining showed that acidic vesicle organelles increased in the NSC23766 group. AO staining showed that apoptotic bodies increased after NSC23766 treatment (**Figure 3C**). The TUNEL staining results showed that the apoptosis of Rac1 inhibitor-treated RMS cells increased compared with that of the control group (*P* < 0.05, **Figure 3D**). The expression level of Active Rac1, Bax, Caspase3, and Cleaved-PARP protein in the NSC23766 group was significantly higher than that in the normal untreated group, whereas the Bcl-2 protein in the NSC23766 group was significantly lower than that in the normal untreated group (P < 0.05 and P < 0.001, **Figure 3E**). NSC27366 could promote apoptosis in the RH30 and RD cells as shown in the flow cytometry analysis. After adding CQ and BafA-1, the apoptosis rate was lower than that of the NSC23766 group (*P* < 0.001, **Figure 3F**). Similarly, the same results were obtained after performing the Western blot (*P* < 0.05 and *P* < 0.001, **Figures 4A, B, E**), immunofluorescence, MDC and AO staining (**Figure 4C**), TUNEL staining (*P* < 0.05, **Figure 4D**), and flow cytometry analysis (P < 0.05 and P < 0.001, **Figure 4F**) after the addition of a Cdc42 inhibitor (ZCL278). All obtained results demonstrated that GEFT affected the autophagy and apoptosis of RMS through Rac1/Cdc42 signaling.

### Rac1/Cdc42 Can Regulate the Expression of mTOR

**Figure 5:**
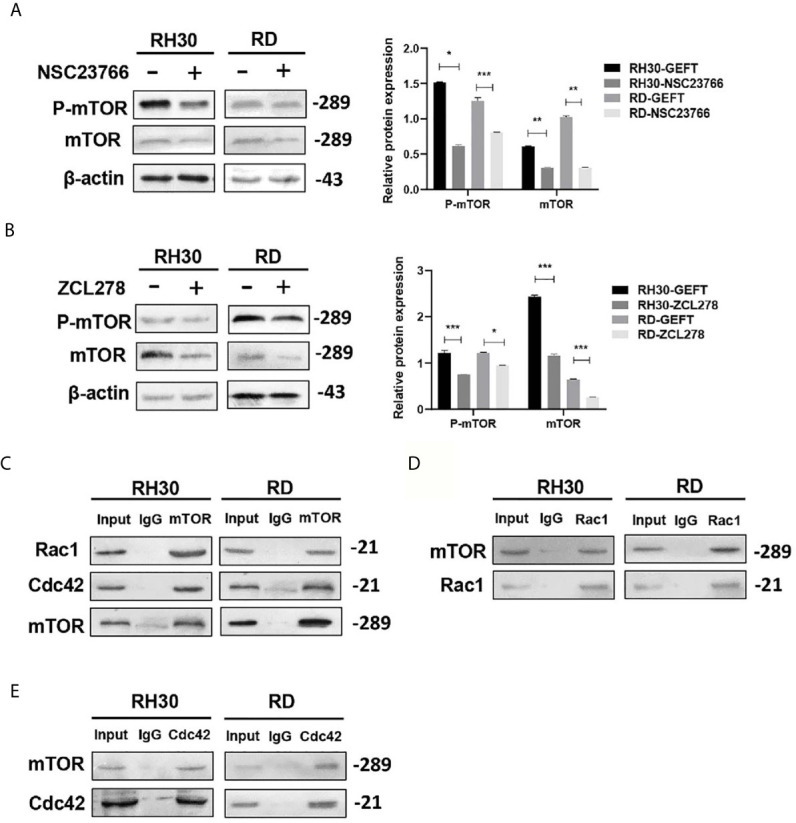
mTOR can be regulated by Rac1/Cdc42. **(A, B)** The Rac1 **(A)** and Cdc42 **(B)** inhibitors were added to the RMS cells. p-mTOR and mTOR were detected by Western blot, and β-actin was used as a control. **(C)** The Rac1 and Cdc42 antibodies were used to pull down mTOR by using the co-immunoprecipitation assay. **(D, E)**. Rac1 **(D)** and Cdc42 **(E)** were pulled down using the mTOR antibody, and the results were examined by Western blot. ***P < 0.001, **P < 0.01, *P < 0.05.

Some studies have shown that Rac1 can form a complex with mTOR to promote its transport to the plasma membrane (23). During intrauterine growth restriction, Rac1 and Cdc42 were positively correlated with mTOR (24). We added Rac1 and Cdc42 inhibitors to detect the expression of mTOR and study whether Rac1 and Cdc42 regulate mTOR in RMS. The results of the Western blot showed that the inhibition of Rac1 (*P* < 0.05, *P* < 0.01, and *P* < 0.001; **Figure 5A**) and Cdc42 (*P* < 0.05 and *P* < 0.001, **Figure 5B**) could reduce the expression of mTOR and p-mTOR. Furthermore, Co-IP was utilized to confirm whether mTOR was sensitive to Rac1 or Cdc42 from the RMS cell lysate, with the control IP performed with nonrelated IgG; the results showed that this was the case, except for the control IgG IP (**Figure 5C**). Reverse validation showed that the presence of mTOR was also detected in the Rac1/Cdc42 antibody co-immunoprecipitated complex (**Figures 5D, E**).

### GEFT Inhibits Autophagy and Apoptosis Through mTOR in RMS Cells

**Figure 6:**
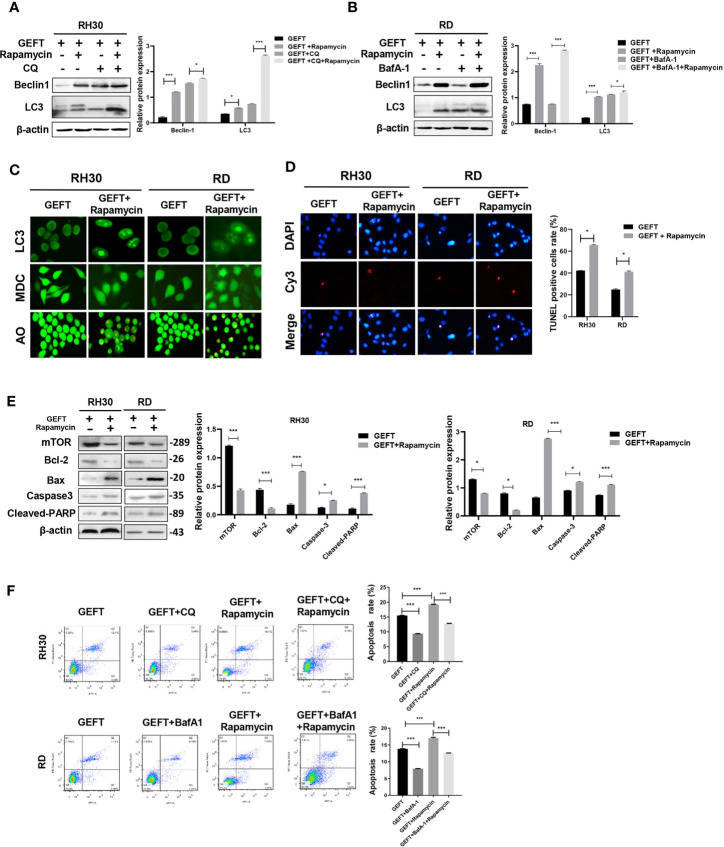
Inhibiting mTOR-promoting autophagy and apoptosis in RMS cells of overexpressing GEFT. **(A)** Western blotting was used to detect the expression of Beclin1 and LC3 protein in RH30 cells transfected with GEFT, GEFT+Rapamycin, GEFT+CQ, and GEFT+CQ+ Rapamycin. **(B)** Western blotting was used to detect the expression of Beclin1 and LC3 protein in RD cells transfected with GEFT, GEFT+Rapamycin, GEFT+BafA-1, and GEFT+BafA-1+Rapamycin. **(C)** The representative images of RH30 and RD cells transfected with GEFT and GEFT+Rapamycin were detected by immunofluorescence, MDC and AO staining. **(D)** The apoptosis of RH30 and RD cells transfected with GEFT and GEFT+Rapamycin was detected by TUNEL. **(E)** Western blotting was used to detect the expression of mTOR, Bax, Bcl-2, Caspase-3, and Cleaved-PARP protein in RH30 and RD cells transfected with GEFT and GEFT+ Rapamycin. **(F)** Flow cytometry was used to detect the apoptosis of RH30 cells and GEFT-transfected RH30 cells in GEFT, GEFT+CQ, GEFT+ Rapamycin and GEFT+ Rapamycin +CQ groups. Flow cytometry was used to detect the apoptosis of RD cells and GEFT-transfected RD cells in GEFT, GEFT+BafA-1, GEFT+Rapamycin and GEFT+ Rapamycin+BafA-1 groups. ***P < 0.001, *P < 0.05.

In addition, in order to continue to explore whether mTOR is involved in autophagy and apoptosis of RMS cells. A mTOR inhibitor (Rapamycin) was added to RMS cells and RMS cells stably transfected with GEFT lentivirus. The results showed that the expression of Beclin1 and LC3 protein in the Rapamycin group was significantly higher than that in the normal untreated group, and the expression levels of Beclin1 and LC3 protein in the Rapamycin group also increased after adding CQ and BafA-1 (*P* < 0.001 and *P* < 0.001, **Figures 6A, B**). In addition, immunofluorescence showed that the dot pattern of LC3 fluorescence was clearly observed in the Rapamycin group. MDC staining showed that acidic vesicle organelles increased in the Rapamycin group. AO staining showed that apoptotic bodies increased after Rapamycin treatment (**Figure 6C**). The TUNEL staining results showed that the apoptosis of mTOR inhibitor-treated RMS cells increased compared with that of the control group (*P* < 0.05, **Figure 6D**). The expression of mTOR, Bax, Caspase3, and Cleaved-PARP protein in the Rapamycin group was significantly higher than that in the normal untreated group, whereas the Bcl-2 protein in the Rapamycin group was significantly lower than that in the normal untreated group (*P* < 0.001 and *P* < 0.001, **Figure 6E**). Rapamycin could significantly promote apoptosis in the RH30 and RD cells as shown in the flow cytometry analysis; after adding CQ and BafA-1, the apoptosis rate was lower than that of the Rapamycin group (*P* < 0.001, **Figure 6F**).

### GEFT–Rac1/Cdc42–mTOR Inhibits Autophagy and Apoptosis in the Xenograft Model

**Figure 7:**
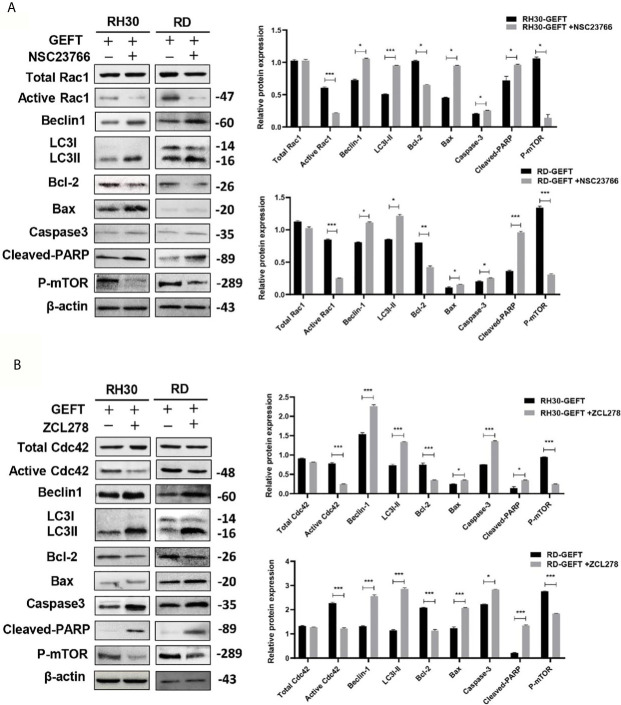
GEFT-mediated Rac1 and Cdc42 inhibit the expression levels of autophagy- and apoptosis-related proteins in transplanted tumor tissues. **(A, B)** Western blot was used to detect the expression of Total Rac1, Active Rac1, Total Cdc42, Active Cdc42, p-mTOR, Beclin1, LC3, Bax, Bcl-2, caspase-3, and cleaved-PARP in RH30 and RD cells transfected with the GEFT group after the addition of Rac1 **(A, B)** Cdc42 inhibitors. ***P < 0.001, **P < 0.01, *P < 0.05.

Previous results revealed that GEFT–Rac1/Cdc42 accelerated tumor growth in mice (9). Finally, we investigated whether GEFT–Rac1/Cdc42 could inhibit tumor autophagy and apoptosis *in vivo*. We examined the tumor tissues of the RH30+GEFT, RH30+GEFT+NSC23766 and RH30+GEFT+ZCL278 groups, respectively. After inhibiting Rac1, the Beclin1, LC3, Caspase-3, Cleaved-PARP and Bax proteins were upregulated, whereas the Active Rac1 and Bcl-2 and p-mTOR proteins were downregulated compared with the GEFT overexpression group (*P* < 0.05, *P* < 0.01, and *P* < 0.001; **Figure 7A**). Simultaneously, after suppressing Cdc42, we found similar results to those for Rac1 (*P* < 0.05, *P* < 0.01, and *P* < 0.001; **Figure 7B**).

**Figure 8:**
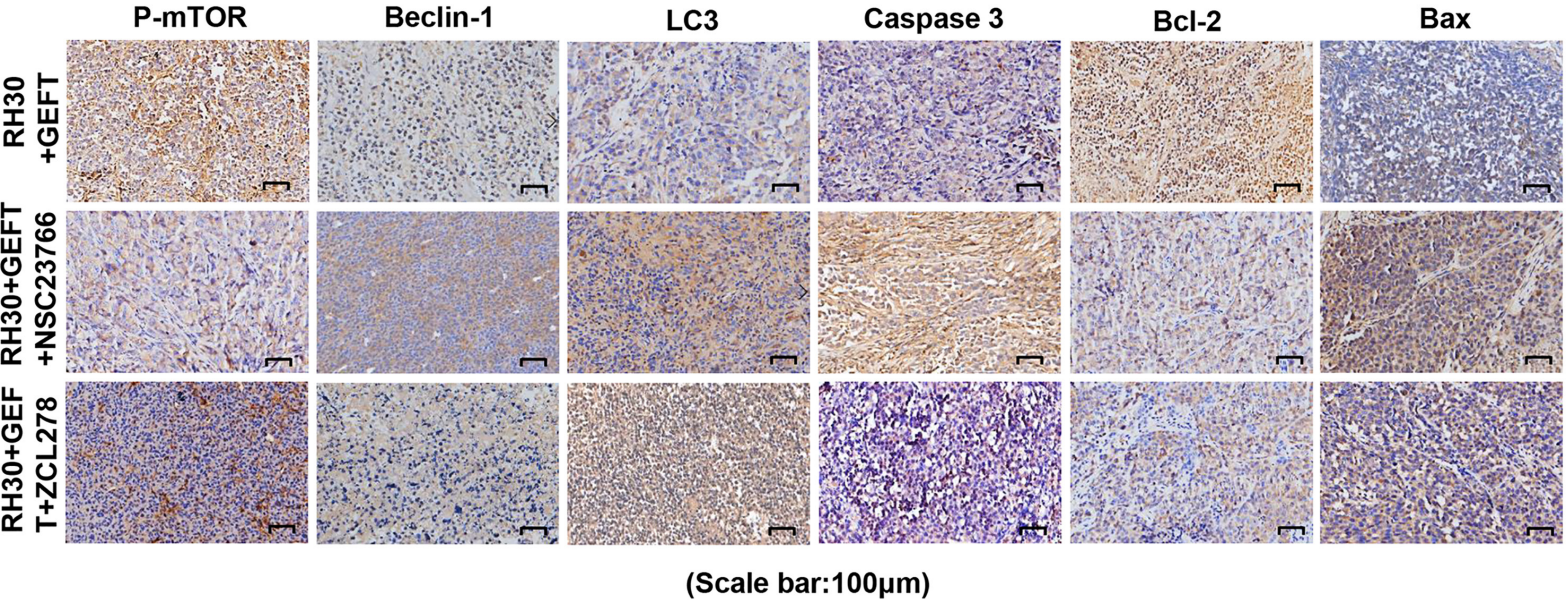
GEFT-mediated Rac1 and Cdc42 inhibit the expression levels of autophagy- and apoptosis-related proteins in transplanted tumor tissues. The expression levels of p-mTOR, Beclin1, LC3, Caspase-3, Bcl-2 and Bax in RH30 transplanted tumor tissues were detected by immunohistochemistry in the RH30+GEFT, RH30+GEFT + NSC23766, and RH30+GEFT + ZCL278 groups. A representative image is provided.

Next, the expression of proteins in the xenograft tumors was assessed using IHC. The results showed that compared with GEFT overexpression group, the addition of Rac1 and Cdc42 inhibitors could increase the expression of Beclin1, LC3, Caspase-3 and Bax, and decreased the p-mTOR and Bcl-2 protein expression (**Supplementary Tables S8**). The representative p-mTOR, Beclin1, LC3, Bax, Caspase-3, and Bcl-2-stained images are shown in **Figure 8**. The RD cell xenograft tumor protein expression was also examined. The results were approximately the same as for the abovementioned RH30 cell xenograft results (**Supplementary Table S9**), the representative p-mTOR, Beclin1, LC3, Bax, Caspase-3, and Bcl-2-stained images are shown in **Supplementary Figure S1**. Overall, these data demonstrated that the GEFT–Rac1/Cdc42–mTOR pathway inhibited autophagy and apoptosis.

## Discussion

Autophagy is an important factor in causing cancer, maintaining tumor stem cells, and resisting malignant tumors. Beclin1 mediates autophagy initiation, and LC3 is a specific marker of autophagy. mTOR regulates the lysosomal reformation and termination of autophagy. In our studies, we confirmed that Beclin1 and LC3 were expressed at high levels in RMS and that mTOR was expressed at low levels in RMS. Beclin1 expression has been shown to be significantly correlated with patient survival in gastric cancer (25) and non-Hodgkin’s lymphoma (26). LC3 expression is positively correlated with clinical stage in oral squamous cell carcinoma (27). We compared multivariate pathological parameters with Beclin1 and LC3 protein expression and found that tumor diameter was positively correlated with Beclin1 protein expression, whereas the high expression of LC3 protein was associated with the clinical stage of the RMS patients.

Apoptosis is a complex and proactive process of cell death controlled by multiple genes. Apoptosis is closely related to the maintenance, atrophy, and inflammation of body’s normal physiological activities. However, defects in apoptosis can lead to an imbalance between cell proliferation and death in the body and cause cancer (28). The Bcl-2 protein plays a central role as a protector of apoptosis, helping cancer cells escape cell death (29). Bax, a key regulator of the mitochondrial apoptotic pathway, accumulates at different focal points on the mitochondrial surface, undergoes conformational changes, and mediates the release of cytochrome c, leading to cell death (30). In our study, we found a high level of Bcl-2 expression and a low level of Bax expression in RMS.

Studies have shown that apoptosis and autophagy possess the same set of regulatory proteins and common upstream signaling components (31, 32). Beclin1 interacts with the Bcl-2 family of anti-apoptotic proteins through the BH3 domain (33), which is well established as a proapoptotic protein (34). Caspase-mediated Beclin1 cleavage inhibits autophagy and promotes S1-induced apoptosis of ovarian cancer cells (35). BMP4 promotes hepatocellular carcinoma proliferation through JNK1-mediated autophagic activation of Bcl-2 phosphorylation (36). ABT-737 induces autophagy through Bax-independent mechanisms and disrupts the binding of Beclin1 to anti-apoptotic Bcl-2 family members (37). In the present study, Bcl-2, Bax, Beclin1, LC3, caspase-3, and Cleaved-PARP were selected for autophagic and apoptotic experiments.

The present study found that Rac1/Cdc42 was highly expressed in RMS, and the IHC results showed that the protein expression was homogeneous in all tumor regions. The autophagy- and apoptosis-related proteins in the RMS cells were downregulated after GEFT overexpression, indicating that GEFT had a positive effect on regulating autophagy and apoptosis. Notably, autophagy- and apoptosis-associated proteins were upregulated and homogeneous in cells overexpressing GEFT after the inhibition of Rac1 and Cdc42. This result was consistent with the inhibition of apoptosis by Rac1 and Cdc42 (38).

Rac1 and Cdc42 are involved in myoblast transformation, and they play an important role in muscle tumors (39). Rac1 inhibition is critical for autophagic flux during starvation and other potential stimuli (40). Rac1 can compete with LC3 for interaction with Armus, preventing it from properly recruiting autophagosomes (41). We found that Beclin1 and LC3 protein expression increased after Rac1 was inhibited in our study. Studies have shown that the activation and expression of Rac1 affect the survival prognosis of many tumor diseases. The status of Rac1-GTP was significantly related to increased mortality and risk of recurrence from breast cancer (42). A high expression level of Rac1 was related to disease-free survival and prolonged survival in lung cancer patients (43). In our study, we revealed that the increased expression of Rac1 was related to the site of tumorigenesis, which affected the survival prognosis of RMS patients. In addition, our study has also confirmed that Cdc42 regulates autophagy.

Studies have shown that p-mTOR is overexpressed in various tumors and is closely related to cancer metastasis and prognosis (44, 45). mTOR plays a key role in regulating cancer cell apoptosis and autophagy (38). mTOR regulates the transport of amino acid transporters in human trophoblasts by mediating Rac1 and Cdc42 (46). mTOR-mediated autophagy regulates the apoptosis induced by diquat (47). Our experimental results demonstrated that p-mTOR was highly expressed in RMS and that the inhibition of mTOR in RMS cell lines promoted apoptosis and autophagy, and this finding was consistent with the idea that mTOR inhibited apoptosis and autophagy in renal carcinoma (48). Rac1 binds directly to mTOR and mediates mTORC2 and mTORC1 localization on specific membranes (49). Rac1 can control cell growth through mTOR signaling (50). S6K1 is activated by the Cdc42–mTOR pathway during retinoic acid-dependent neural differentiation to promote cell growth (51). In our study, mTOR and p-mTOR were reduced by Western blot experiments with the addition of Rac1 and Cdc42 inhibitors. Moreover, Co-IP experiments demonstrated that the presence of mTOR was detected in the co-immunoprecipitation complex of Rac1 and Cdc42 antibodies. Our results verified that mTOR may play a role through the Rac1/Cdc42–mTOR signaling pathway.

NSC23766 is considered to be a specific inhibitor of Rac1. NSC23766 effectively inhibits Rac1 binding and activation through Rac-specific GEF Trio or Tiam1, and does not interfere with closely related Cdc42 or RhoA binding or activation (52). ZCL278 has become a selective Cdc42 small molecule regulator, which directly binds to Cdc42 and inhibits its function (53). Rapamycin is a specific inhibitor of mTOR protein, which binds to intracellular receptor FKBP-12 to form a complex and then directly acts on the FRB domain of mTOR to inhibit protein activity (54). In the past few years, Rapamycin has been developed as a treatment for a variety of cancers. Rapamycin combined with short-term radiotherapy can be used in the treatment of rectal cancer (55). According to another study, autophagy is closely related to mTOR signal pathway inhibitors in the treatment of glomerulonephritis (56). Rapamycin reduced T cell failure caused by bladder cancer, and the prevalence of PD-1 expression of T cells decreased significantly (57). In this experiment, NSC23766, ZCL278 and Rapamycin can promote apoptosis and autophagy of RMS cells, it can be used as a potential drug to kill RMS cells.

**Figure 9:**
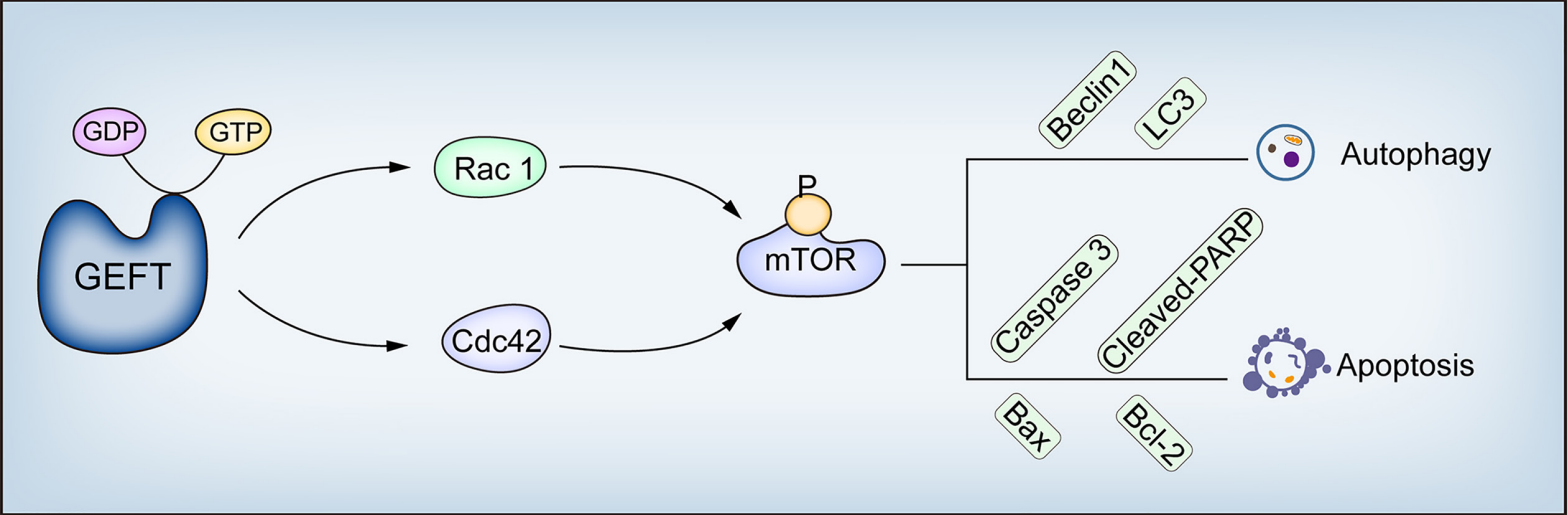
GEFT mechanism diagram in RMS. GEFT inhibits autophagy and apoptosis through the Rac1/Cdc42–mTOR pathway.

Our results suggest that GEFT modulates the Rac1/Cdc42-mTOR pathway to inhibit autophagy and apoptosis in RMS. This study describes the molecular mechanism of GEFT inhibiting autophagy and apoptosis in RMS; the expression of Rac1, Cdc42, p-mTOR, Beclin1, LC3, Bax, and Bcl-2; and the relationship among the clinical pathological parameters in RMS, which has revealed and enriched the understanding of GEFT’s carcinogenic mechanism (**Figure 9**). Understanding autophagy and apoptosis in RMS can reveal new targets and pathways to improve the treatment of drug-resistant tumors.

## Data Availability Statement

The original contributions presented in the study are included in the article/**Supplementary Material**, further inquiries can be directed to the corresponding authors.

## Ethics Statement

The studies involving human participants were reviewed and approved by The First Affiliated Hospital of Shihezi University. The patients/participants provided their written informed consent to participate in this study. The animal study was reviewed and approved by The First Affiliated Hospital of Shihezi University. Written informed consent was obtained from the individual(s) for the publication of any potentially identifiable images or data included in this article.

## Author Contributions

CSL and FL designed the research. CSL, LS, ZL, LM, GX, HZ, and JH carried out experiment and analyzed and interpreted data. CSL and CXL wrote the manuscript. All authors contributed to the article and approved the submitted version.

## Funding

This work was supported by grants from the National Natural Science Foundation of China (81660441, 81960485 and 81460404) and Science and Technology Development Project of Xinjiang Production and Construction Corps (2018AB033) and Beijing Natural Science Foundation (Grant/Award Number: 7194272).

## Conflict of Interest

The authors declare that the research was conducted in the absence of any commercial or financial relationships that could be construed as a potential conflict of interest.
