# Supplementary material for: GEFT Inhibits Autophagy and Apoptosis in Rhabdomyosarcoma via Activation of the Rac1/Cdc42-mTOR Signaling Pathway
Source: Front Oncol. 2021 Jun 18;11:656608. doi: 10.3389/fonc.2021.656608 (PMC8252888; doi:10.3389/fonc.2021.656608)
Supplement: Supplementary file 1 [file DataSheet_1.docx]

**Supplementary Table. S1**. Relationship between Rac1 protein expression and clinicopathological parameters in RMS.

| **Variables** | N | Rac 1 | | *X^2^* value | P value |
| --- | --- | --- | --- | --- | --- |
|  |  | −/1+ (*n*) | 2+/3+ (*n*) |  |  |
| **Gender** |  |  |  |  |  |
| Male | 33 | 16 | 17 |  |  |
| Female | 29 | 17 | 12 | 0.637 | 0.425 |
| **Age (years)** |  |  |  |  |  |
| ≤ 5 | 17 | 9 | 8 |  |  |
| > 5 | 45 | 24 | 21 | 0.001 | 0.978 |
| **Ethnicity** |  |  |  |  |  |
| Han | 29 | 13 | 16 |  |  |
| Other minorities | 33 | 20 | 13 | 0.361 | 0.548 |
| **Tumor diameter** |  |  |  |  |  |
| ≤ 5 cm | 27 | 13 | 14 |  |  |
| > 5 cm | 34 | 19 | 15 | 3.673 | 0.159 |
| **Histologic type** |  |  |  |  |  |
| ARMS | 18 | 6 | 12 |  |  |
| ERMS | 29 | 18 | 11 | 3.673 | 0.159 |
| PRMS | 8 | 4 | 4 |  |  |
| **Location** |  |  |  |  |  |
| Head and neck | 19 | 5 | 14 |  |  |
| Extremities and trunk | 22 | 14 | 8 | 9.371 | 0.025 |
| Genitourinary tract | 11 | 6 | 5 |  |  |
| Thoracic cavity | 10 | 8 | 2 |  |  |
| **TNM Stage** |  |  |  |  |  |
| I and II | 24 | 13 | 11 | - | 0.278 |
| III and IV | 21 | 10 | 11 |  |  |
| **Lymph node metastasis** |  |  |  |  |  |
| No | 35 | 18 | 17 | - | 0.410 |
| Yes | 10 | 5 | 5 |  |  |
| **Distant metastasis** |  |  |  |  |  |
| No | 32 | 15 | 17 | - | 0.536 |
| Yes | 13 | 8 | 5 |  |  |

Note: Other minorities including Uygur (n = 26), Kazakh (n = 2) and Hui (n = 3) and Zhuang (n = 2).

**Supplementary Table. S2**. Cox proportional hazards model analysis of patients with RMS.

| **Variables** | Univariate analysis | | Multivariate analysis | |
| --- | --- | --- | --- | --- |
|  | HR (95% CI) | P value | HR (95% CI) | P value |
| **Rac1 protein** |  |  |  |  |
| −/1+ | 0.343 (0.134, 0.878) | 0.026 | - | - |
| 2+/3+ | 1 |  | - |  |
| **Gender** |  |  |  |  |
| Male | 0.837 (0.328, 2.136) | 0.710 | 0(0, 961.21) | 0.268 |
| Female | 1 |  | 1 |  |
| **Age (years)** |  |  |  |  |
| ≤ 5 | 0.112 (0.015, 0.841) | 0.033 | 1.13(0, 1.451) | 0.433 |
| > 5 | 1 |  | 1 |  |
| **Ethnicity** |  |  |  |  |
| Han | 0.818 (0.331, 2.022) | 0.664 | 1.63 (0, 3.21) | 0.380 |
| Other minorities | 1 |  | 1 |  |
| **Tumor diameter** |  |  |  |  |
| ＞5 cm | 1.180 (0.476, 2.924) | 0.721 | 0.014 (0, 1.832) | 0.870 |
| ≤5 cm | 1 |  | 1 |  |
| **Histologic type** |  |  |  |  |
| ERMS | 1.295 (0.336, 4.987) | 0.707 | 1 | 0.534 |
| ARMS | 0.325 (0.075, 1.434) | 0.134 | 0.037(0, 1209.45) |  |
| PRMS | 1 |  | 1 |  |
| **Location** |  |  |  |  |
| Head and neck | 3.988(0.768, 20.713) | 0.100 | 1 |  |
| Extremities and trunk | 3.553(0.716, 17.627) | 0.121 | - | - |
| Genitourinary tract | 3.782(0.493, 29.002) | 0.201 | 0.0085 (0,1.181) 3813.758) | 0.38 |
| Thoracic cavity retroperitoneal | 1 |  | 7394(0.001, 5.255) | 0.268 |
| **TNM Stage** |  |  |  |  |
| I and II | 0.066 (0.007, 0.576) |  | - | - |
| III and IV | 1 | 0.014 | - |  |
| **Lymphnode metastasis** |  |  |  |  |
| No | 0.110 (0.01, 1.239) |  | - | - |
| Yes | 1 | 0.074 | - |  |
| **Distant metastasis** |  |  |  |  |
| No | 0.229 (0.032, 1.656) |  | - |  |
| Yes | 1 | 0.144 | - | - |

**Supplementary Table. S3.** Correlation analysis of GEFT and Rac1, Cdc42, p-mTOR Beclin1, LC3, Bax and Bcl-2 protein expression.

| Molecules | | GEFT (%) | | Phi (r) | P |
| --- | --- | --- | --- | --- | --- |
|  |  | Negative | Positive |  |  |
| Rac1 | Negative | 3 (100) | 0 (0) | 1.000 | ＜0.001 |
|  | Positive | 0 (0) | 18 (100) |  |  |
| Cdc42 | Negative | 3 (100) | 0 (0) | 1.000 | ＜0.001 |
|  | Positive | 0 (0) | 18 (100) |  |  |
| p-mTOR | Negative | 1 (33.3) | 0 (0) | 0.548 | 0.012 |
|  | Positive | 2 (66.7) | 18 (100) |  |  |
| Beclin1 | Negative | 0 (0) | 6 (33.3) | -0.258 | 0.237 |
|  | Positive | 3 (100) | 12 (66.7) |  |  |
| LC3 | Negative | 0 (0) | 11 (61.1) | -0.428 | 0.05 |
|  | Positive | 3 (100) | 7 (38.9) |  |  |
| Bax | Negative | 0 (0) | 7 (38.9) | -0.289 | 0.186 |
|  | Positive | 3 (100) | 11 (61.1) |  |  |
| Bcl-2 | Negative | 2 (66.7) | 0 (0) | 0.795 | 0.001 |
|  | Positive | 1 (33.3) | 18 (100) |  |  |

| Tissue type | N | Beclin1 | | χ^2^ | *P* | LC3 | | χ^2^ | *P* |
| --- | --- | --- | --- | --- | --- | --- | --- | --- | --- |
|  |  | Negative | Positive |  |  | Negative | Positive |  |  |
| RMS | 62 | 17 (27.4) | 45 (72.6) | 5.350 | 0.021 | 19 (30.6) | 43 (69.3) | 4.092 | 0.043 |
| ARMS | 18 | 2 (11.1) | 16 (88.9) |  |  | 4 (22.2) | 14 (77.8) |  |  |
| ERMS | 29 | 13 (44.9) | 16 (55.1) |  |  | 11 (37.9) | 18 (62) |  |  |
| PRMS | 8 | 1 (12.5) | 7 (87.5) |  |  | 3 (37.5) | 5 (62.5) |  |  |
| Other | 7 | 1 (14.3) | 6 (85.7) |  |  | 1 (14.3) | 6 (85.7) |  |  |
| Control | 20 | 0 (0) | 20 (100) |  |  | 1 (5) | 19 (95.0) |  |  |

**Supplementary Table. S4.** Expression of Beclin1 and LC3 protein in different types of RMS and normal muscle tissue.

Others represent nonspecific types of RMS. Control represents normal muscle tissue.

**Supplementary Table. S5.** Relationship between Beclin1 protein expression and clinicopathological parameters in RMS.

| **Variables** | N | Beclin1 | | *X^2^* value | P value |
| --- | --- | --- | --- | --- | --- |
|  |  | −/1+ (*n*) | 2+/3+ (*n*) |  |  |
| **Gender** |  |  |  |  |  |
| Male | 33 | 15 | 18 | 3.064 | 0.08 |
| Female | 29 | 7 | 22 |  |  |
| **Age (years)** |  |  |  |  |  |
| ≤ 5 | 17 | 7 | 10 | 0.565 | 0.767 |
| > 5 | 45 | 15 | 30 |  |  |
| **Ethnicity** |  |  |  |  |  |
| Han | 29 | 9 | 20 | 0.518 | 0.772 |
| Other minorities | 33 | 13 | 20 |  |  |
| **Tumor diameter** |  |  |  |  |  |
| ≤ 5 cm | 27 | 13 | 14 | 4.040 | 0.044 |
| > 5 cm | 34 | 8 | 26 |  |  |
| **Histologic type** |  |  |  |  |  |
| ARMS | 18 | 3 | 15 |  |  |
| ERMS | 29 | 13 | 16 | 4.558 | 0.102 |
| PRMS | 8 | 4 | 4 |  |  |
| **Location** |  |  |  |  |  |
| Head and neck | 19 | 8 | 11 |  |  |
| Extremities and trunk | 22 | 8 | 14 | 1.899 | 0.623 |
| Genitourinary tract | 11 | 2 | 9 |  |  |
| Thoracic cavity | 10 | 4 | 6 |  |  |
| **TNM Stage** |  |  |  |  |  |
| I and II | 18 | 4 | 14 | - | 1 |
| III and IV | 14 | 3 | 11 |  |  |
| **Lymph node metastasis** |  |  |  |  |  |
| No | 27 | 7 | 20 | - | 0.769 |
| Yes | 5 | 0 | 5 |  |  |
| **Distant metastasis** |  |  |  |  |  |
| No | 25 | 6 | 19 | - | 0.912 |
| Yes | 7 | 1 | 6 |  |  |

Note: Other minorities including Uygur (n = 26), Kazakh (n = 2) and Hui (n = 3) and Zhuang (n = 2).

**Supplementary Table. S6.** Relationship between LC3 protein expression and clinicopathological parameters in RMS.

| **Variables** | N | LC3 | | *X^2^* value | P value |
| --- | --- | --- | --- | --- | --- |
|  |  | −/1+ (*n*) | 2+/3+ (*n*) |  |  |
| **Gender** |  |  |  |  |  |
| Male | 33 | 20 | 13 | 0.014 | 0.906 |
| Female | 29 | 18 | 11 |  |  |
| **Age (years)** |  |  |  |  |  |
| ≤ 5 | 17 | 13 | 4 | 2.275 | 0.131 |
| > 5 | 45 | 25 | 20 |  |  |
| **Ethnicity** |  |  |  |  |  |
| Han | 29 | 17 | 12 | 1.781 | 0.411 |
| Other minorities | 33 | 21 | 12 |  |  |
| **Tumor diameter** |  |  |  |  |  |
| ≤ 5 cm | 27 | 19 | 8 | 1.345 | 0.246 |
| > 5 cm | 34 | 19 | 15 |  |  |
| **Histologic type** |  |  |  |  |  |
| ARMS | 18 | 13 | 5 |  |  |
| ERMS | 29 | 18 | 11 | 1.247 | 0.536 |
| PRMS | 8 | 4 | 4 |  |  |
| **Location** |  |  |  |  |  |
| Head and neck | 19 | 11 | 8 |  |  |
| Extremities and trunk | 22 | 13 | 9 | 1.824 | 0.610 |
| Genitourinary tract | 11 | 6 | 5 |  |  |
| Thoracic cavity | 10 | 8 | 2 |  |  |
| **TNM Stage** |  |  |  |  |  |
| I and II | 18 | 15 | 3 | - | 0.027 |
| III and IV | 14 | 6 | 8 |  |  |
| **Lymph node metastasis** |  |  |  |  |  |
| No | 27 | 12 | 15 | - | 0.645 |
| Yes | 5 | 3 | 2 |  |  |
| **Distant metastasis** |  |  |  |  |  |
| No | 25 | 12 | 13 | - | 0.576 |
| Yes | 7 | 3 | 4 |  |  |

Note: Other minorities including Uygur (n = 26), Kazakh (n = 2) and Hui (n = 3) and Zhuang (n = 2).

| Tissue type | N | Beclin1 | | χ^2^ | *P* | LC3 | | χ^2^ | *P* |
| --- | --- | --- | --- | --- | --- | --- | --- | --- | --- |
|  |  | Negative | Positive |  |  | Negative | Positive |  |  |
| RMS | 30 | 14 (46.7) | 16 (53.3) | 8.101 | 0.004 | 2 (6.7) | 28 (93.3) | 15.469 | 0.001 |
| ARMS | 12 | 6 (50.0) | 6 (50.0) |  |  | 1 (8.3) | 11 (91.7) |  |  |
| ERMS | 15 | 7 (46.7) | 8 (53.3) |  |  | 1 (6.7) | 14 (93.3) |  |  |
| PRMS | 3 | 1 (33.3) | 2 (66.7) |  |  | 0 (0) | 3 (100) |  |  |
| Control | 15 | 0 (0) | 15 (100) |  |  | 10 (66.7) | 5 (33.3) |  |  |

**Supplementary Table. S7.** Expression of Bax and Bcl-2 protein in different types of RMS and normal muscle tissue.

Control represents normal muscle tissue.

| Protein Molecule | N | RH30+GEFT(%) | | RH30+GEFT+NSC23766(%) | | RH30+GEFT+ZCL278(%) | |
| --- | --- | --- | --- | --- | --- | --- | --- |
|  |  | Negative | Positive | Negative | Positive | Negative | Positive |
| Active Rac1 | 5 | 0 (0) | 5 (100) | 4 (80) | 1 (20) | 0 (0) | 5 (100) |
| Active Cdc42 | 5 | 0 (0) | 5 (100) | 0 (0) | 5 (100) | 4 (80) | 1 (20) |
| P-mTOR | 5 | 0 (0) | 5 (100) | 2 (40) | 3 (60) | 2 (40) | 3 (60) |
| Beclin 1 | 5 | 2 (40) | 3 (60) | 0 (0) | 5 (100) | 0 (0) | 5 (100) |
| LC3 | 5 | 2 (40) | 3 (60) | 0 (0) | 5 (100) | 0 (0) | 5 (100) |
| Caspase 3 | 5 | 2 (40) | 3 (60) | 0 (0) | 5 (80) | 0 (0) | 5 (100) |
| Bcl-2 | 5 | 0 (0) | 5 (100) | 2 (40) | 3 (60) | 2 (40) | 3 (60) |
| Bax | 5 | 1 (0) | 4 (80) | 0 (0) | 5 (100) | 0 (0) | 5 (100) |

**Supplementary Table S8.** Expression of different molecular proteins in transplanted tumor tissue of RH30 group.

**Supplementary Table S9.** Expression of different molecular proteins in transplanted tumor tissue of RD group.

| Protein Molecule | N | RH30+GEFT(%) | | RH30+GEFT+NSC23766(%) | | RH30+GEFT+ZCL278(%) | |
| --- | --- | --- | --- | --- | --- | --- | --- |
|  |  | Negative | Positive | Negative | Positive | Negative | Positive |
| Beclin 1 | 5 | 3 (60) | 2 (40) | 0 (0) | 5 (100) | 0 (0) | 5 (100) |
| LC3 | 5 | 3 (60) | 2 (40) | 0 (0) | 5 (100) | 0 (0) | 5 (100) |
| Caspase 3 | 5 | 0 (0) | 5 (100) | 1 (20) | 4 (80) | 2 (40) | 3 (60) |
| Bcl-2 | 5 | 1 (20) | 4 (80) | 0 (0) | 5 (100) | 0 (0) | 5 (100) |
| Bax | 5 | 0 (0) | 5 (100) | 2 (40) | 3 (60) | 2 (40) | 3 (60) |


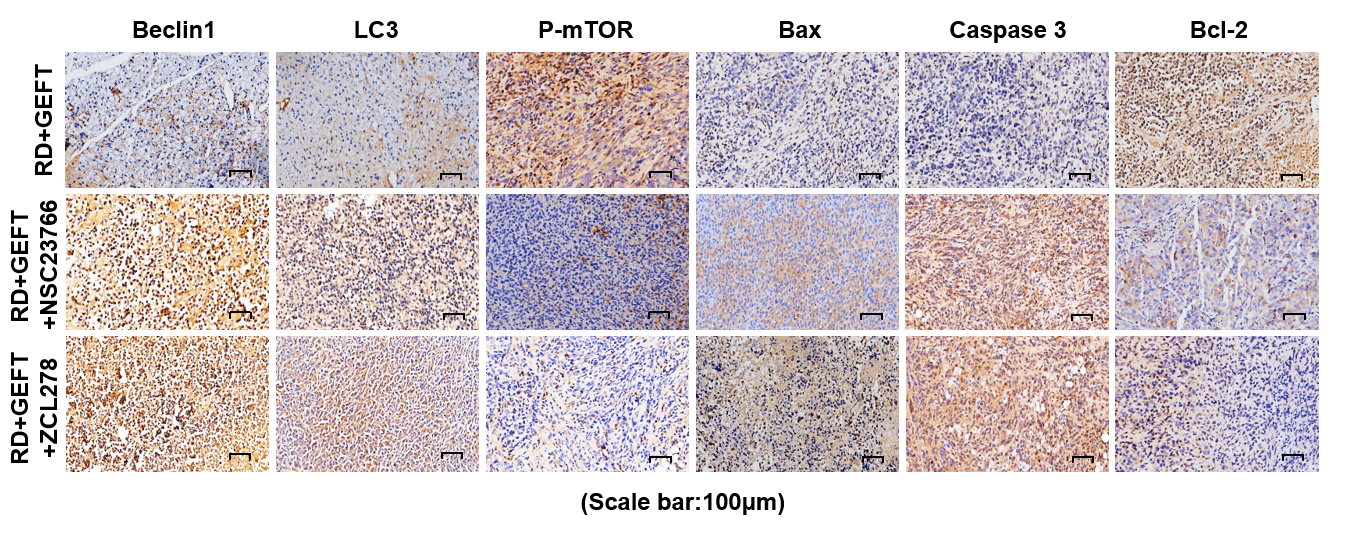
**Supplementary FIGURE 1.** GEFT-mediated Rac1 and Cdc42 inhibit the expression levels of autophagy- and apoptosis-related proteins in transplanted tumour tissues. The expression levels of Beclin1, LC3, p-mTOR, Bax, Caspase-3, and Bcl-2 in RD transplanted tumour tissues were determined using IHC. A representative image is provided.
